# Some Remarks on Hernia

**Published:** 1908-10-24

**Authors:** 


### Surgery.

### SOME REMARKS ON HERNIA.

### III. BASSINI'S OPERATION FOR RADICAL CURE OF AN INGUINAL HERNIA.

Many operations are in vogue at the present day for the radical cure of an inguinal hernia. In most of them the same objects are aimed at, namely, the removal of the sac after ligature of the neck, and the formation of a new and stronger inguinal canal. Within limits the result obtained is equally successful whatever particular method is chosen, and it follows that the important point in the operation is simplicity of technique; for the simpler the technique of the operation, the more rapidly it can be performed, and the less interference there is with the natural condition of the part.

In this sense Bassini's operation, of all the methods described, fulfils the required conditions most completely. An incision is made about three inches long, parallel to the external abdominal ring, that is to say, almost parallel to Poupart's ligament. Its lower extremity should be brought down to the lower end of the external abdominal ring. The aponeurosis of the external oblique should next be exposed. It is essential to do this thoroughly in order that the field of operation may be clearly defined. The aponeurosis is then split up in the direction of its fibres as high as the position of the internal ring. This may either be done with a pair of scissors or by passing a grooved director underneath the muscle and running a knife along it. A Spencer-Wells clip should be put on each edge in order that it may be subsequently identified and to facilitate the introduction of the deep stitches. The cord is now exposed.

It is often a matter of some difficulty to be able to recognise among its constituents the sac of the hernia. This is best done by passing it between the thumb and fingers and looking for the opaque white line which is formed by its doubled edge. A clip should be placed on the edge as soon as it is found, and the sac can then be isolated from the rest of the cord as far as the internal abdominal ring. In an acquired case, so called, the sac is of varying length and terminates in a blind extremity at its apex. Much more frequently, however, than text-books give one to expect the sac is of the congenital variety, and one then finds a peritoneal tube leading from the internal abdominal ring right down to the testicle. In some cases it is difficult to isolate the sac, even when its edge has been found, on account of its extreme thinness: this is particularly likely to be the case in very young patients, or in those who have been wearing a truss for any length of time. One is told that truss-pressure tends to produce a chronic inflammatory condition in the sac and to make it thick. The writer may have been unfortunate, but it is his experience that this is quite a rare event.

As soon as the sac is isolated it must be opened. This is an invariable rule in all cases of hernia. It it literally not safe to ligature the sac of a hernia without opening it, since there is no means of knowing whether a portion of small intestine has been included in the ligature or not; and this oversight would have the direst consequences. When the sac is opened its contents, if any, must be replaced in the abdomen by the assistant introducing his finger, which is pushed in until the tip is up against the internal abdominal ring, and kept in that position until a ligature has been applied. If the sac is of tne congenital variety the procedure is essentially the same, except that the lower end of the sac must be freed from the testicle by cutting it all round about a quarter of an inch from the edge of the testicle itself. The free edge attached to the testicle very rarely bleeds, and in this it differs from the edge which is left after the radical operation for the cure of a hydrocele of the tunica vaginalis; but ere this the tunica vaginalis is in a state of chronic congestion, and its vessels are enlarged. Should, however, any hæmorrhage occur, the bleeding points must be picked up and ligatured in the ordinary way. It is quite unnecessary, and even inadvisable, to unite these edges and attempt to form an artificial tunica vaginalis.

The next step is to deal with the inguinal canal. In Bassini's operation, as originally described by him, the conjoined tendon is united to Poupart's ligament by a row of sutures passing through each, and tied *behind* the cord; so that a new posterior wall is formed to the inguinal canal. But an excellent modification is to tie these sutures *in front of* the cord. This small change in the technique has much to recommend it and no drawbacks. It is easier to do, and the operation, therefore, takes less time; and the inguinal canal so produced much more nearly resembles the normal structure than in the official Bassini operation. The fibres of the external oblique are then united in front of the cord, and the wound is sewn up.

Much difference of opinion exists as to the material which should be used to unite the conjoined tendon to Poupart's ligament. Many surgeons use kangaroo-tendon, and the prejudice in favour of its use appears to be widely spread. It is true that it is strong and holds well, but it is difficult to be certain of its asepticity, and most surgeons who use it can recall outbreaks of sepsis among their radical cures, in which no other possible cause could be found to account for it. It is inadvisable to use silkworm gut for buried sutures on account of the stiff ends which have an uncomfortable habit of staying erect, and possibly making their way through the wound. On the whole the best material in the writer's opinion is stout silk, which is quite strong enough and lasting enough for all practical purposes; besides which it ought to be possbile to be sure of its sterility.

This operation has been described in detail because it is at once the simplest and the most efficient. No method which depends upon twisting the sac and suturing it in some unnatural position should be countenanced for a moment.

